# 1,25 Dihydroxyvitamin D_3_ Inhibits TGFβ_1_-Mediated Primary Human Cardiac Myofibroblast Activation

**DOI:** 10.1371/journal.pone.0128655

**Published:** 2015-06-10

**Authors:** Anna Meredith, Seti Boroomand, Jon Carthy, Zongshu Luo, Bruce McManus

**Affiliations:** 1 Centre for Heart Lung Innovation, St. Paul’s Hospital, University of British Columbia, Vancouver, BC, Canada; 2 Department of Pathology and Laboratory Medicine, University of British Columbia, Vancouver, BC, Canada; 3 Ludwig Institute for Cancer Research, Uppsala, Sweden; 4 PROOF Centre of Excellence, Vancouver, BC, Canada; University of Oklahoma Health Sciences Center, UNITED STATES

## Abstract

**Aims:**

Epidemiological and interventional studies have suggested a protective role for vitamin D in cardiovascular disease, and basic research has implicated vitamin D as a potential inhibitor of fibrosis in a number of organ systems; yet little is known regarding direct effects of vitamin D on human cardiac cells. Given the critical role of fibrotic responses in end stage cardiac disease, we examined the effect of active vitamin D treatment on fibrotic responses in primary human adult ventricular cardiac fibroblasts (HCF-av), and investigated the relationship between circulating vitamin D (25(OH)D_3_) and cardiac fibrosis in human myocardial samples.

**Methods and Results:**

Interstitial cardiac fibrosis in end stage HF was evaluated by image analysis of picrosirius red stained myocardial sections. Serum 25(OH)D_3_ levels were assayed using mass spectrometry. Commercially available HCF-av were treated with transforming growth factor (TGF)β_1_ to induce activation, in the presence or absence of active vitamin D (1,25(OH)_2_D_3_). Functional responses of fibroblasts were analyzed by *in vitro* collagen gel contraction assay. 1,25(OH)_2_D_3_ treatment significantly inhibited TGFβ_1_-mediated cell contraction, and confocal imaging demonstrated reduced stress fiber formation in the presence of 1,25(OH)_2_D_3_. Treatment with 1,25(OH)_2_D_3_ reduced alpha-smooth muscle actin expression to control levels and inhibited SMAD2 phosphorylation.

**Conclusions:**

Our results demonstrate that active vitamin D can prevent TGFβ1-mediated biochemical and functional pro-fibrotic changes in human primary cardiac fibroblasts. An inverse relationship between vitamin D status and cardiac fibrosis in end stage heart failure was observed. Collectively, our data support an inhibitory role for vitamin D in cardiac fibrosis.

## Introduction

Heart failure (HF) represents a growing health concern worldwide, with overall incidence rates of 1-2%, and 12% of individuals over 80 years [1] affected by the condition. Although treatment and management programs for HF patients have improved, one-year post-diagnosis mortality remains between 25–40% [2]. The social impact of cardiac failure is further compounded by the significant, and growing, resource utilization for HF management [1]. Demographic projections indicate HF prevalence will increase three-fold by 2050, implying that HF will place an ever larger burden on national health services, and emphasizing the urgent need for improvements in disease prevention to curtail spiraling economic and social costs.

Increased fibrosis and aberrant wound healing response resolution are well characterized pathophysiological hallmarks of HF. Strategies to limit the persistent pro-fibrotic response observed throughout compensatory cardiac remodeling in HF may provide novel therapeutic strategies to stem the burden of this condition. Cardiac fibroblasts are the most numerous cell type in the heart. They contribute to extracellular matrix (ECM) development and maintenance through collagen synthesis and remodeling, providing the structural, mechanical and electrical integrity essential for the efficient translation of cardiac myocyte contraction into cardiac output. Their role in preservation of cardiac structure in both health and disease is essential for maintenance of end organ perfusion throughout the body. The critical role of cardiac fibroblasts in preserving cardiac function in response to injury highlights their potential as an attractive therapeutic target in efforts to modulate fibrosis in the setting of HF. In settings of injury, fibroblasts are activated, and undergo transformation to myofibroblasts, the latter being cell populations characterized by their increased synthetic and contractile properties. This fibrotic response is essential in maintaining the structure of the heart and preserving cardiac function in response to injury, however unresolved fibrotic remodeling, can lead to increased residual interstitial fibrosis and result in myocardial stiffness, imperfect electrical propagation and myocyte disarray. Transforming growth factor (TGF) β is a potent activator of fibroblasts, known to induce myofibroblastic activation and induce increased collagen deposition and wound contraction [3]. TGFβ’s role in fibrosis and fibroproliferative disorders is well described in the biomedical literature. It is a key mediator of fibrosis in myocardial injury [4] and has been shown to contribute to unresolved cardiac pro-fibrotic remodeling [5, 6] as observed in HF. Strategies to inhibit TGFβ are increasingly being investigated with the objective of developing novel HF therapeutics. Abrogation of TGFβ signaling using neutralizing antibodies or oral pharmacological inhibitors has shown promising results in animal models of cardiac remodeling and HF [7, 8].

Vitamin D, an endogenously produced hormone, has garnered increasing attention for its potential role in cardiovascular (CV) health [9, 10]. Current guidelines define deficiency as circulating levels below 20ng/ml [50nmol/l]; insufficiency as circulating levels of 21-29ng/ml [50-75nmol/l], and sufficiency as ≥30ng/ml [75nmol/l] [11]. Studies of vitamin D status have indicated that a large proportion of the population worldwide may be vitamin D deficient [12, 13, 14], and that significant reductions in mortality and healthcare expenditures could be achieved if mean population serum vitamin D levels were increased [15, 16]. The vitamin D receptor (VDR) is expressed throughout the human CV system [17] and clinical data have provided some evidence of a protective effect of vitamin D on cardiac remodeling and HF survival. Characterization of VDR knockout mice has demonstrated increased cardiac fibrosis [18], however cardiomyocyte-specific VDR deletion does not result in increased interstitial cardiac fibrosis [19], suggesting that non-myocyte VDR contributes to the observed fibrotic phenotype. Data from murine renal, hepatic and pulmonary fibroblasts has provided evidence for an inhibitory role of active vitamin D in the TGFβ-mediated fibrotic response in these cells, through a variety of mechanisms. Further, treatment with the VDR agonist paricalcitol reduced myocardial fibrosis and improved cardiac structure and function post-LAD ligation in a mouse model of myocardial infarction [20]. Together these data provide mechanistic clues commensurate with the epidemiological evidence supporting vitamin D’s role in CV health, however direct effects of vitamin D treatment on human cardiac cells have not been previously reported. We investigated the hypothesis that vitamin D could inhibit TGFβ induced myofibroblastic activation in human cardiac fibroblasts *in vitro*, and that vitamin D status would correlate with extent of interstitial cardiac fibrosis in patients with end stage HF. A wealth of data exist on the pro-fibrotic milieu present in myocardial remodeling, and targeting these progressive remodeling processes will be essential to attenuation of HF burden.

## Materials and Methods

### Human Subjects and Ethics Statement

Patients evaluated for cardiac transplantation for end stage HF at St. Paul’s Hospital, Vancouver, British Columbia between January 2005 and August 2009 were approached, and those providing written consent were enrolled in the study. Pre-transplant blood serum samples were collected from patients on the transplant list and myocardial samples were obtained from explanted native hearts at the time of transplantation. Twenty-three patients had serum samples collected within one month of transplantation, and myocardial tissue available for analysis, and were selected for inclusion. Subject demographics are presented in Table 1. All work was performed according to the guidelines of the Declaration of Helsinki and was approved by the Human Research Ethics Board of the University of British Columbia and the Providence Health Care Research Ethics Board.

**Table 1:** Study Cohort Demographic Information.

	Vitamin D deficient	Vitamin D insufficient	Vitamin D sufficient	p-value
**n**	7	11	5	
**Age (years)**	41.6 (14.7)	55.5 (12.1)	61.4 (3.0)	**0.033**
**Female (%)**	1 (14)	2 (18)	2 (40)	0.525
**Serum 25(OH)_2_D_3_ (nmol/L)**	39.9 (10.4)	63.9 (7.3)	89.7 (19.0)	**<0.0001**
**Etiology of heart failure**				
**Ischemic**	1 (14.3)	7 (63.6)	3 (60)	0.103
**Nonischemic dilated cardiomyopathy**	6 (85.7)	3 (27.3)	2 (40)	0.050
**Arrhythmogenic right ventricular cardiomyopathy**	0 (0)	1 (9.1)	0 (0)	0.565

Data expressed as mean (± SD) or n (%), p-values calculated using one-way analysis of variance for continuous variables and chi-square test for categorical variables.

### Vitamin D Assay

Circulating levels of 25(OH)_2_D_3_ were assayed by the Clinical Laboratory at St. Paul's Hospital (Vancouver, BC) using an API 5000 System mass spectrometer (AB SCIEX, Framingham, MA) connected to a 20AD Dual Binary UFLC (Shimadzu, Columbia, MD). 100μL of patient serum was precipitated with 350μL of a 95:5 acetonitrile:methanol solution containing 36ng/mL 25-hydroxy vitamin—D3-26,26,26,27,27,27-d6 internal standard. 2 MRM's were monitored for VitD3 and VitD2, 1 MRM was monitored for the internal standard. Quantitation was performed using a 1 point calibration curve for each MRM, linear-through-zero. Precision evaluation for this assay was performed using a modification of the CLSI EP-5A2 protocol. Quintuplicate analysis of pooled human samples (n = 20–30 per level) with clinically relevant levels were analyzed over 5 days. Within-run coefficient of variation (CV), between-run CV and total CV for 25(OH)D3 at 64.8 nmol/L was 3.7%, 0.4% and 3.7% respectively; at 119.1 nmol/L was 2.1%, 2.8% and 3.5% respectively. The within-run, between-run and total CV for 25(OH)D2 at 35.8 nmol/L was 3.7%, 5.8% and 6.9%, respectively.

### Histology

Formalin fixed paraffin embedded (FFPE) transmural lateral left ventricular free wall (LVFW) myocardial samples were taken from explanted HF hearts and sectioned at 4 microns. Sections were deparaffinized and stained with hematoxylin and eosin for evaluation of general morphology, and picrosirius red for collagen staining and quantification of fibrosis.

### Image Analysis

Collagen area of picrosirius red stained sections was quantified by colour segmentation using ImagePRO Plus (Media Cybernetics, Bethesda, MD). Briefly, 5 random fields per case were taken at X20 magnification using a digital spot camera (Microspot; Nikon, Tokyo, Japan). Images were captured by a single observer blinded to the vitamin D status of each patient, collagen area was calculated as a percentage of tissue area per field, excluding non-tissue area and blood vessels. Sections were evaluated by an experienced cardiac pathologist to exclude infarct and peri-infarct areas, and to ensure all measures represented interstitial fibrosis.

### Cell Culture

Commercially available primary adult ventricular human cardiac fibroblasts (HCF-av) were used for all experiments (Catalog #6310, Sciencell, CA, USA—ordered in 2012). Cells were maintained in a humidified incubator at 37°C with 5% CO2 and used for experiments between passages 3–7 at 70% confluence. Cells were cultured on poly-L-lysine coated culture plates in fibroblast medium (FM) (Sciencell) containing 10% FBS, growth supplements and penicillin/streptomycin, as per manufacturer’s instructions. 1,25 dihydroxyvitamin D_3_ (1,25(OH)_2_D_3_) (Sigma, Oakville, ON) was reconstituted in 95% ethanol. All experiments used Transforming Growth Factor (TGF)β_1_ (BioLegend, San Diego, CA) at 10ng/ml and 1,25(OH)_2_D_3_ at 1μM. 95% ethanol was used as vehicle control. Protein was extracted at 24 and 48 hours following treatment.

### Confocal Imaging

Cells cultured in chamber slides were fixed in 4% paraformaldehyde for 30 minutes, permeabilized with 0.1% triton X-100 for 20 minutes, and blocked for 30 minutes with 1% bovine serum albumin (BSA) in phosphate buffered saline (PBS). F-actin was visualized using a 1:500 dilution of phalloidin-Alexa fluor 594 (Invitrogen, Burlington, ON), incubated for 30 minutes at room temperature. Cells were coverslipped with VectaShield mounting medium containing DAPI (Vector Laboratories, Burlingame, CA) and images were captured using a Leica AOBS SP2 confocal microscope as described previously [21]. Images were processed using Volocity software (Improvision, Coventry, UK).

### Immunoblotting

Total protein was extracted from treated HCF-av cells as described previously [21], separated using 4–15% gradient sodium dodecylsufate-polyacrylamide gel electrophoresis and transferred to a nitrocellulose membrane. Membranes were blocked for 1 hour in 5% milk/TBS Tween 20 (TBST) and incubated overnight at 4°C with primary antibody in 5% milk/TBST. The following primary antibody dilutions were used; αSMA (1:500), CYP24A1 (1:500), DDR2 (1:500), VDR (1:500) (Santa Cruz Biotechnology, Santa Cruz, CA), pSMAD2 (1:1000) (Cell Signaling, Danvers, MA). Following three washes in TBST, HRP-conjugated secondary antibody (Santa Cruz) at a concentration of 1:2000 in 5% milk/TBST was added for 1 hour at room temperature. GAPDH (1:500) (Santa Cruz) was used as a loading control. Antibody binding was visualized with enhanced chemiluminescence detection (Thermo Fisher Scientific, Waltham, MA). Images were captured using the Chemigenius2 system (Syngene, Frederick, MD) and band intensities were calculated using ImageJ [22].

### Collagen Gel Contraction Assay

Collagen gel contraction assays were performed as described previously by our group [21]. In brief, 12-well culture plates were coated in 1% bovine serum albumin (Sigma) for 24 hours at 37°C to provide a non-adherent surface for collagen gels. HCF-av were grown to confluency, trypsinized and seeded at a density of 5 × 10^4^ cells per well in treatment media containing 0.5mg/mL type I collagen solution (BD Biosciences, San Jose, CA). The collagen cell suspension was mixed by pipetting, and 1mL total volume per well was plated. Collagen gel contraction was monitored over 96 hours, and gels were imaged at 24, 48, 72 and 96 hour time points. Gel areas were calculated from digital images using Image-Pro Plus (Media Cybernetics, Bethesda, MD), and contraction calculated as a percentage of surface area relative to untreated control gels.

### Proliferation Assay

Cellular proliferation was assayed using 5-bromo-2-deoxyuridine (BrdU) incorporation. HCF-av were seeded at a density of 1 × 10^4^ cells per well in 96-well plates. After 24 hours, media was replaced with fresh treatment media containing TGFβ_1_ at 10ng/mL, 1,25(OH)_2_D_3_ at 1μM, or both. Cells were incubated for 48 hours and then labeled with BrdU for 24 hours. BrdU incorporation was evaluated using the Cell Proliferation Assay Kit (Cell Signaling) as per manufacturer’s directions. Absorbance was read at 450nm using a VersaMax microplate reader (Molecular Devices, Sunnyvale, CA).

### Data Analysis

Statistical analysis was performed using GraphPad Prism version 5.01 for Windows (Graphpad Software, La Jolla, CA). Serum 25(OH)_2_D_3_ and myocardial fibrosis, BrdU absorbance, and Western blot densitometry data were analyzed ANOVA with post hoc Bonferroni multiple comparison correction. Collagen gel contraction assays were assessed using two-way ANOVA with Bonferroni post tests. Categorical data for all experiments were compared by the chi-square test. For all experiments, significance was set at P<0.05.

## Results

### Primary Cardiac Fibroblasts Express a Functional Vitamin D Receptor

**Fig 1:**
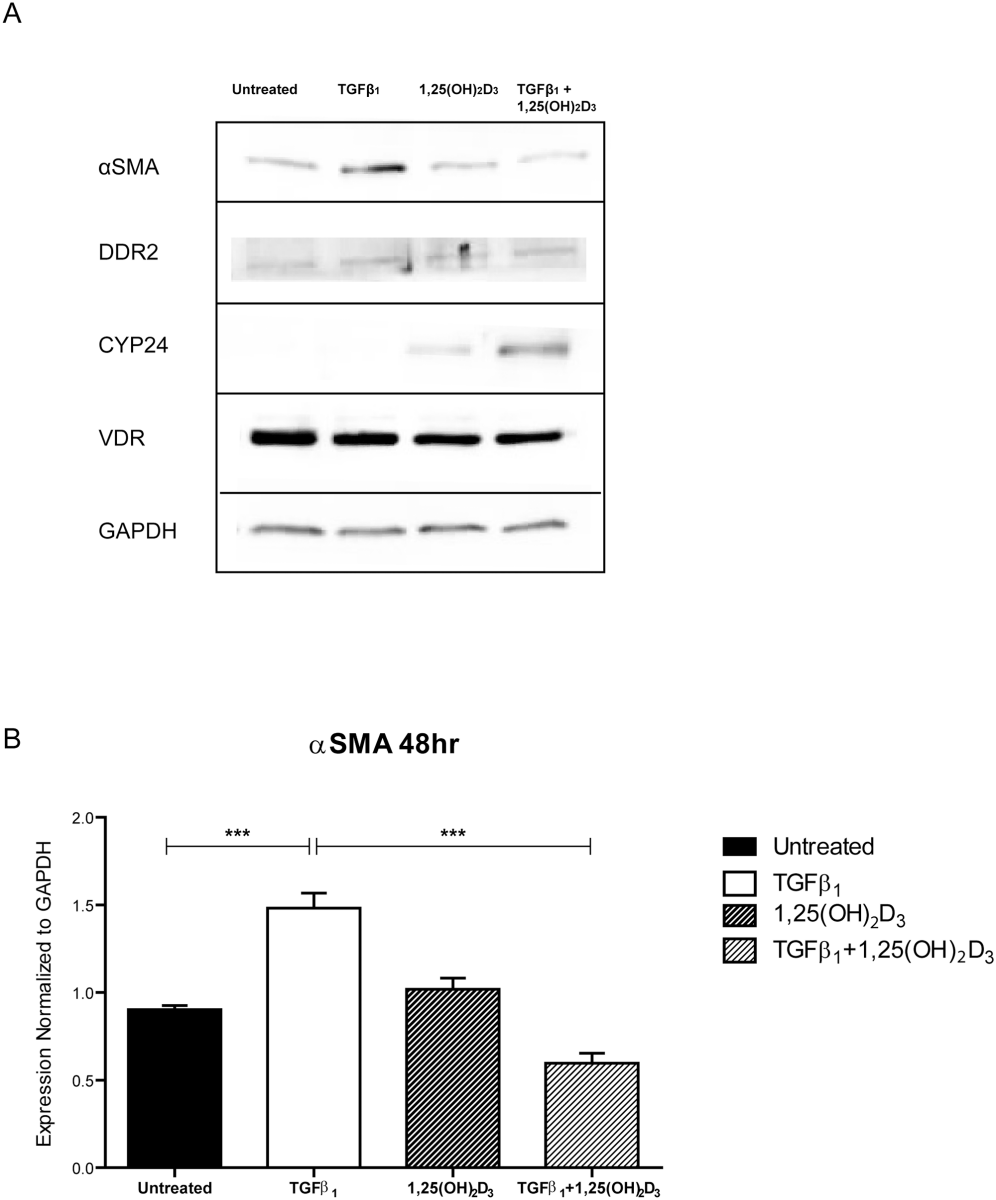
Vitamin D treatment inhibits expression of TGFβ_1_-mediated α-smooth muscle actin. A) Representative Western blot of cells 48 hours after treatment. Expression of the discoidin domain receptor 2 (DDR2) is present in human primary adult ventricular cardiac fibroblasts (HCF-av). CYP24 expression was upregulated 48 hours after treatment with 1,25(OH)_2_D_3_±TGFβ_1_. Expression of α-smooth muscle actin (αSMA) was upregulated 48 hours after treatment with TGFβ_1_, and significantly reduced with 1,25(OH)_2_D_3_ co-treatment. B) Densitometry data generated from Western blots of HCF-av cells 48 hours after treatment with TGFβ_1_±1,25(OH)_2_D_3_, and normalized to GAPDH. All data represent mean±SEM. p-values were calculated using one way analysis of variance with a Bonferroni multiple comparison test. *p<0.05, ***<p<0.001.

We confirmed the primary cells we used in our *in vitro* experiments were fibroblasts by Western blot for discoidin domain receptor 2 (DDR2). Western blot for VDR was used to confirm primary cardiac fibroblast expression of the vitamin D receptor. To confirm the validity of our model system, we examined whether our HCF-av cells expressed a functional VDR. CYP24 is a well known VDR target gene, which is upregulated in the presence of the VDR ligand 1,25(OH)_2_D_3_. Treatment of HCF-av cells with 1,25(OH)_2_D_3_ resulted in upregulation of CYP24, consistent with previous biomedical literature on vitamin D signaling (Fig 1A).

### 1,25(OH)_2_D_3_ Inhibits TGFβ_1_-mediated Myofibroblast Activation of Primary Human Cardiac Fibroblasts

**Fig 2:**
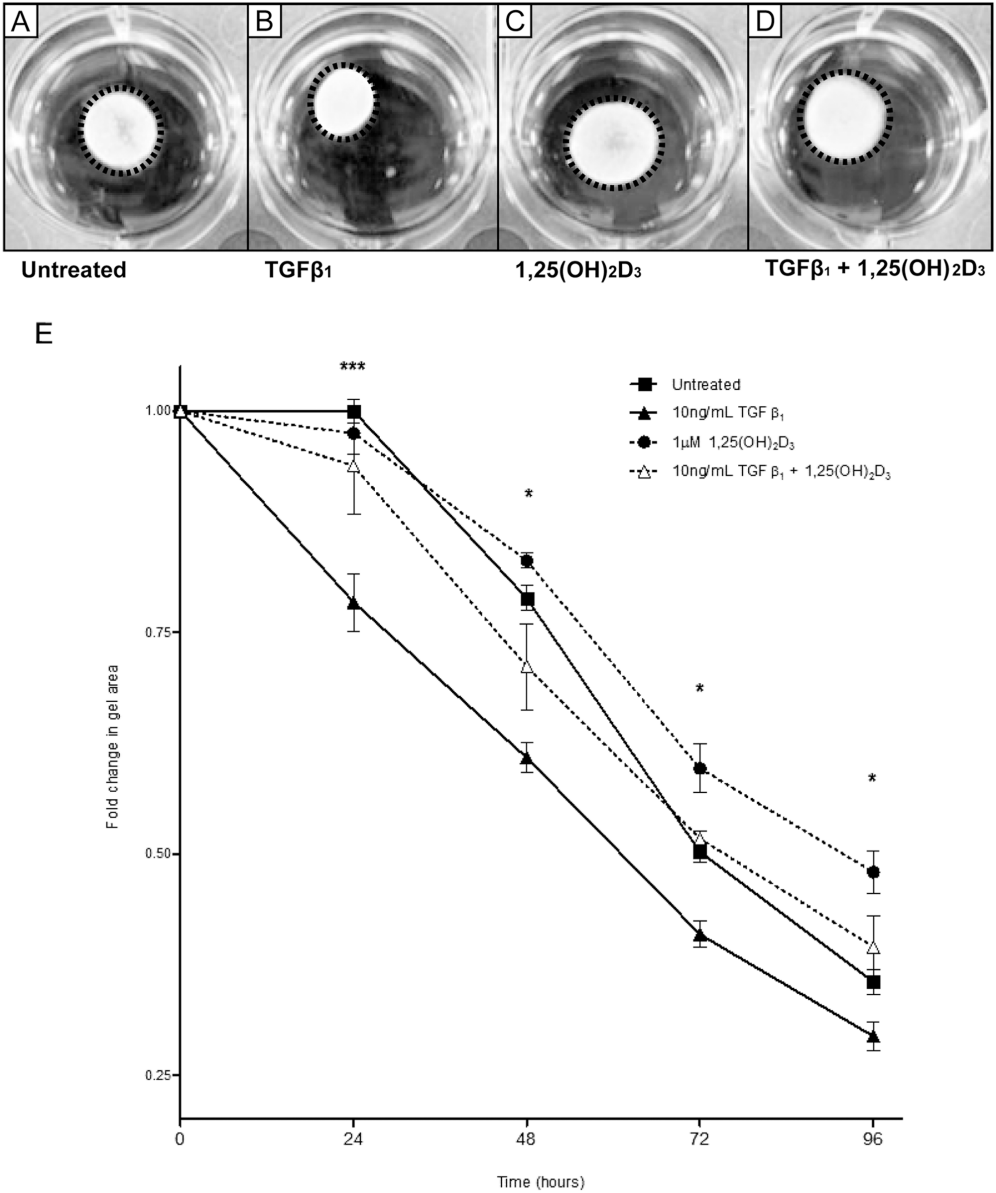
Vitamin D inhibits TGFβ_1_-mediated myofibroblast contraction. Representative images of 48 hour timepoint from collagen gel contraction assay are shown in A-D. A) Untreated HCF-av; B) HCF-av treated with TGFβ_1_; C) HCF-av treated with 1,25(OH)_2_D_3_; D) HCF-av treated with TGFβ_1_ + 1,25(OH)_2_D_3_. A time course of gel contraction over 96 hours is shown in (E). Active vitamin D treatment significantly inhibited TGFβ_1_-induced gel contraction at all time points post-treatment. All data points represent mean ± SEM. p-values calculated using two way repeated measures analysis of variance with Bonferroni multiple comparison test. *p<0.05, ***p<0.001. F and G) Confocal images of HCF-av at 48 hours following treatment. Stress fibers were stained with phalloidin. HCF-av treated with TGFβ_1_(F) demonstrate increased presence of clearly defined stress fibers (white) as compared with cells treated with TGFβ_1_ + 1,25(OH)_2_D_3_ (G). Scale bar: 70μm.

TGFβ_1_ is a potent pro-fibrotic mediator known to induce myofibroblastic activation in cardiac fibroblasts. It strongly upregulates the expression of the myofibroblast marker αSMA, and drives a secretory and contractile phenotype in these cells. As such, TGFβ_1_ is a critical mediator of aberrant extracellular matrix deposition and remodeling in fibrotic diseases. We evaluated the expression of αSMA following treatment of HCF-av with TGFβ_1_ in the presence and absence of 1,25(OH)_2_D_3_ to determine whether treatment with active vitamin D could abrogate TGFβ_1_-mediated myofibroblast activation. TGFβ_1_ treatment resulted in significant upregulation of αSMA expression (p<0.05) at 48 hours following treatment. Co-treatment of cells with 1,25(OH)_2_D_3_ reduced αSMA levels to baseline (p<0.001) (Fig 1B). Myofibroblastic activation is characterized by stress fiber formation. Stress fibers incorporate αSMA, allowing myofibroblasts to generate increased contractile force on the matrix surrounding them, and their formation is induced by TGFβ_1_. Confocal imaging of f-actin stained cells following treatment with TGFβ_1_ ± 1,25(OH)_2_D_3_ demonstrated a reduction in stress fiber staining intensity in the presence of active vitamin D_3_ (Fig 2G).

### 1,25(OH)_2_D_3_ Inhibits TGFβ_1-_induced Collagen Gel Contraction

**Fig 3:**
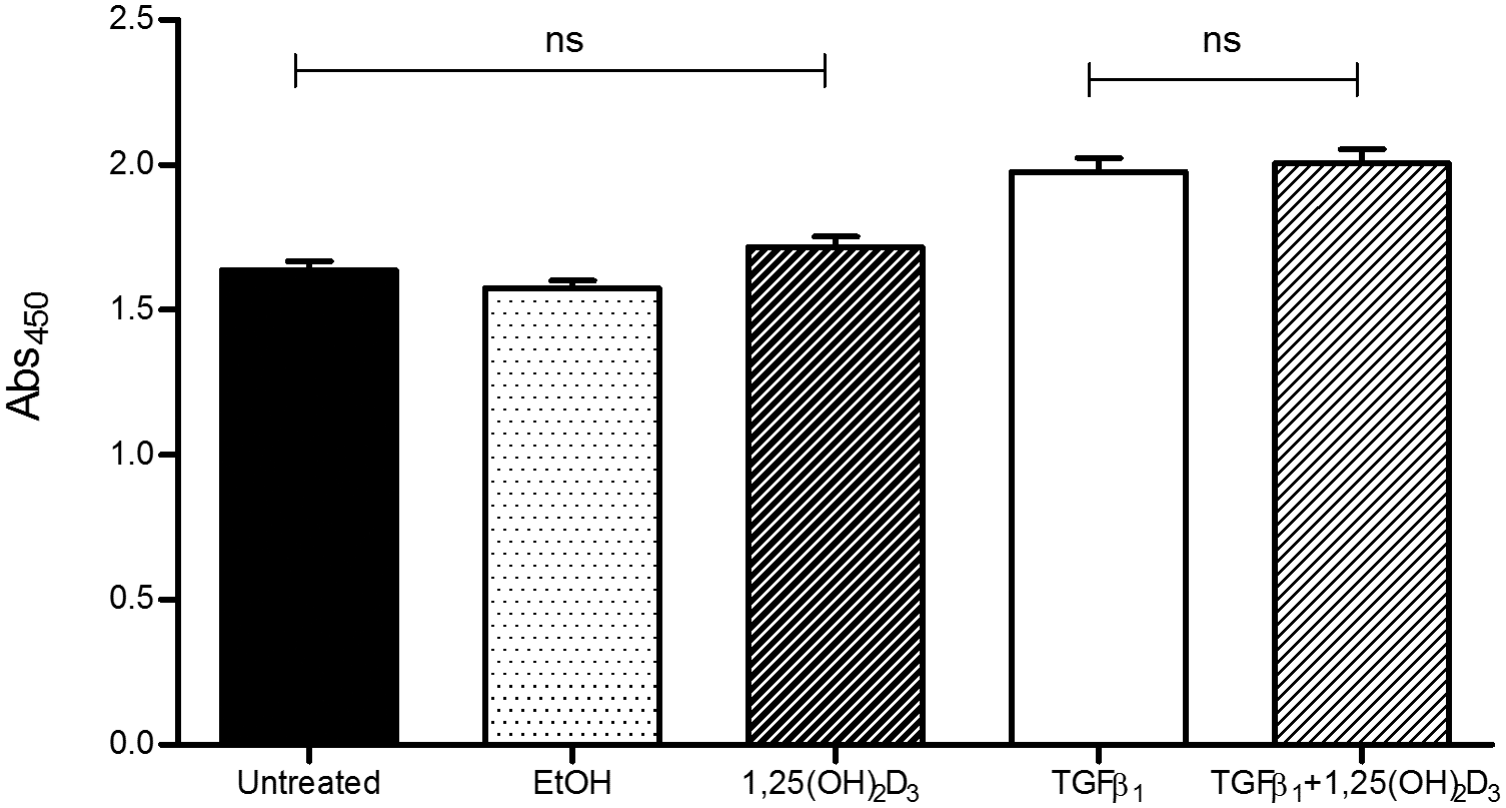
Vitamin D does not inhibit TGFβ_1_-mediated cellular proliferation. Evaluation of proliferation rates in our treatment groups revealed no significant change in cellular proliferation between cells treated with active vitamin D and TGFβ_1_ or TGFβ_1_ alone. Proliferation was increased in the presence of TGFβ_1_. All data represent mean ± SEM. p-values were calculated using one way analysis of variance with a Bonferroni multiple comparison test.

The biochemical changes elicited by TGFβ1 are paralleled by functional changes *in vitro*, mediated by the contractile phenotype of activated myofibroblasts. To examine whether vitamin D can act to inhibit these functional effects we performed a collagen gel contraction assay. Fibroblast contraction, as measured by this model, was significantly increased in cells treated with TGFβ_1_ at all timepoints. Concurrent treatment with TGFβ_1_ + 1,25(OH)_2_D_3_ reduced collagen gel contraction to untreated control levels throughout the 96 hour time course (p<0.05) (Fig 2E). To determine whether the changes in gel contraction we observed resulted from increased cellular proliferation in the presence of TGFβ_1_ we performed a BrdU incorporation proliferation assay. Although TGFβ_1_ significantly increased cell numbers, there was no significant difference in cells co-treated with 1,25(OH)_2_D_3_ (Fig 3) suggesting the observed differences in gel contraction were not a function of cell number.

### Active Vitamin D Inhibits TGFβ_1_-induced SMAD2 Phosphorylation

**Fig 4:**
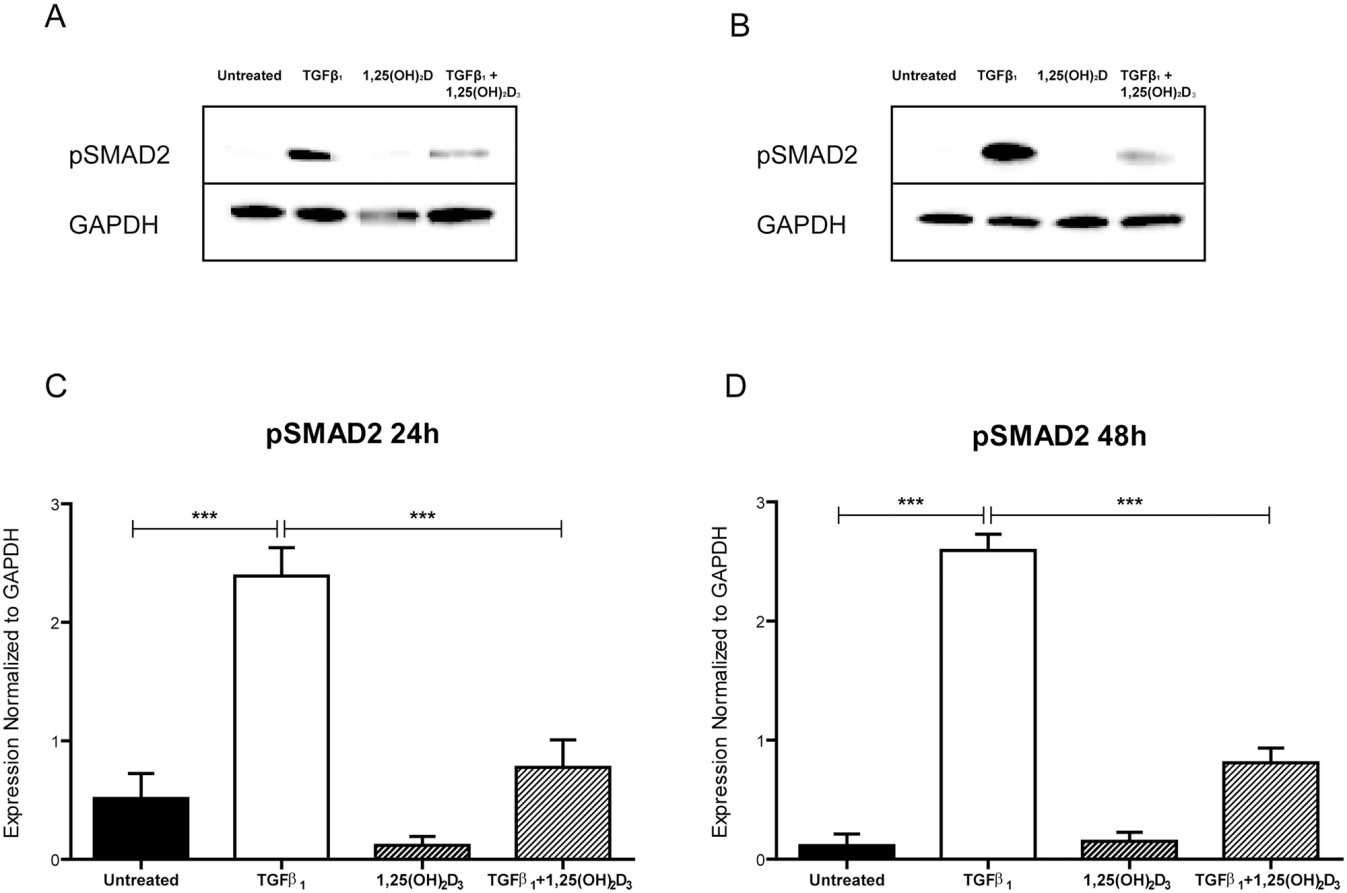
Vitamin D reduces TGFβ_1_-mediated phosphorylation of SMAD2. Representative Western blot images of HCF-av treated for 24 hours (A) and 48 hours (B) with TGFβ_1_ in the presence and absence of 1,25(OH)_2_D_3_, which demonstrate a reduction in pSMAD2 with active vitamin D treatment. Densitometry of Western blot data shows significantly increased phosphorylation of SMAD2 at both 24 hours (C) and 48 hours (D) following treatment with TGFβ_1_, which is significantly reduced with co-treatment with 1,25(OH)_2_D_3_. All data represent mean ± SEM. p-values were calculated using one way analysis of variance with a Bonferroni multiple comparison test. ***p<0.001, ****p<0.0001.

TGFβ_1_ signals via SMAD dependent and independent pathways. We investigated whether vitamin D abrogated the SMAD dependent pathway by evaluating differences in SMAD2 phosphorylation in the presence of active vitamin D by Western blot. Treatment of cells with TGFβ_1_ significantly increased pSMAD2 levels in HCF-av cells both 24 and 48 hours after treatment (Fig 4) (p<0.0001). Addition of 1,25(OH)_2_D_3_ significantly reduced pSMAD2 levels at both timepoints (p<0.001 relative to TGFβ_1_). Our results indicate that active vitamin D can significantly inhibit TGFβ_1_-mediated SMAD2 phosphorylation and in this way diminish TGFβ_1_ downstream signaling events and gene transcription.

### Circulating 25(OH)D_3_ Is Inversely Correlated with Myocardial Fibrosis in End-stage Heart Failure

**Fig 5:**
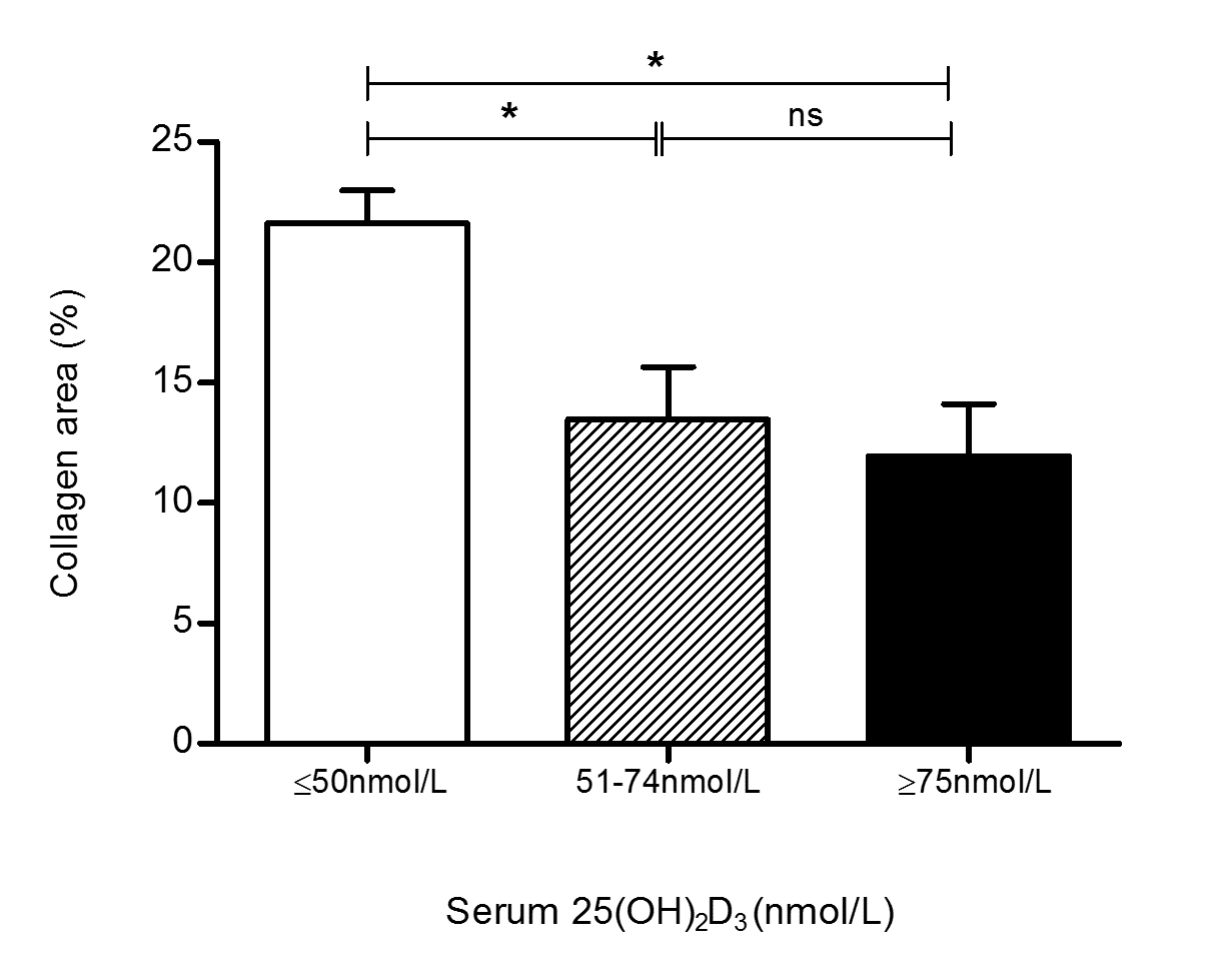
Circulating levels of vitamin D are positively associated with myocardial fibrosis in heart failure patients. Collagen area as a fraction of total myocardial tissue area was significantly higher in patients with vitamin D deficiency as compared with vitamin D insufficient or sufficient patients. There was no difference in collagen area in patients without vitamin D deficiency. All data represent mean ± SEM. p-values were calculated using one way analysis of variance with a Bonferroni multiple comparison test. *p<0.05.

Vitamin D status and area of interstitial fibrosis in explanted hearts was evaluated in 23 end-stage HF patients summarized in Table 1. Our analysis demonstrated a reduction in myocardial interstitial collagen area in patients with higher circulating 25(OH)_2_D_3_ (Fig 5). The data were analyzed in order to group patients by current guideline levels of vitamin D, revealing that HF patients deficient in vitamin D (circulating 25(OH)_2_D_3_ levels <50nmol/L) had significantly higher levels of cardiac fibrosis than patients with circulating levels above 50nmol/L (p<0.05). There was no difference in fibrotic area between patients with insufficient vitamin D levels (50-74nmol/L) and those with vitamin D sufficiency (≥75nmol/L), suggesting that there is a threshold level above which additional benefit is not observed.

## Discussion

In this study we present mechanistic evidence of an anti-fibrotic effect of vitamin D in human cardiac fibroblasts and have shown that circulating vitamin D levels are negatively associated with myocardial fibrosis in end-stage HF. Our *in vitro* data indicate that vitamin D may inhibit TGFβ_1_-mediated SMAD2 phosphorylation, and can reduce both biochemical and functional measures of myofibroblastic activation.

The role of vitamin D in the human heart has remained poorly defined, although the presence of vitamin D signaling machinery within the human myocardium implies a direct role for this hormone in cardiac physiology. We identified expression of a functional VDR in primary human cardiac fibroblasts, as demonstrated by upregulation of its known target gene CYP24 in the presence of the VDR ligand 1,25(OH)_2_D_3_. We induced myofibroblastic activation of primary cardiac fibroblasts with TGFβ_1_, and demonstrated the expected increases in protein expression of αSMA, matrix remodeling proteins, and associated augmentation of contractile function measured using a gel contraction assay. These characteristic myofibroblastic features were diminished in the presence of 1,25(OH)_2_D_3_. Epidemiological data suggests a protective role for vitamin D in the setting of HF, however the direct effects of circulating vitamin on cardiac structure or function remain unknown. In light of our *in vitro* data we evaluated explanted end stage HF myocardial tissue to determine whether we could observe any alterations in fibrotic remodeling in relation to vitamin D status. Histological evaluation of interstitial fibrosis demonstrated an inverse relationship between circulating levels of the vitamin D intermediate 25(OH)D_3_ and extent of fibrotic remodeling.

Evidence linking vitamin D to cardiovascular health has accumulated in recent years: numerous epidemiological studies report deficiency as a significant cardiovascular disease (CVD) risk factor, and, as well, vitamin D levels are inversely correlated with known CVD risk factors and with incidence of overt CVD [23, 24, 25]. HF is associated with a high prevalence of vitamin D deficiency, and low levels of vitamin D are in turn correlated with adverse clinical outcomes in this patient population. Low vitamin D levels are associated with elevated BNP, increased hospitalization rates, and higher incidence of all-cause mortality in HF [26].

Data from rodent models of myocardial injury suggest active vitamin D can modulate critical remodeling processes, including cardiac hypertrophy [27] and extracellular matrix remodeling [20, 28]. To date there have been limited data identifying the specific mechanisms by which vitamin D may exert benefit in the setting of human cardiac disease. The effects of vitamin D on myofibroblast activation have been studied in both murine pulmonary and renal interstitial fibroblasts, and demonstrated that vitamin D inhibits the pro-fibrotic effects of TGFβ_1_ in these cells [29, 30]. An extensive body of work has also shown a role for vitamin D in inhibiting hepatic fibrosis [31]. A recent study reported that ligand bound VDR directly interacts with SMAD3 to inhibit SMAD dependent TGFβ signaling in a mouse model of tubulointerstitial renal fibrosis [32]. A similar mechanism was described in the context of systemic sclerosis wherein primary human skin fibroblasts treated with the VDR agonist paricalcitol, ligand-bound VDR associated with phosphorylated SMAD3 to inhibit downstream gene transcription [33]. Our work supports data from these disease models, and indicates that vitamin D can inhibit SMAD dependent TGFβ signaling in cardiac fibroblasts. However, the mechanisms by which vitamin D acts on fibrosis are not fully elucidated. A study on the effects of the vitamin D analog paricalcitol has indicated that the potential mechanism by which the activated VDR exerts its effects is via inhibition of β-catenin mediated gene transcription [34]. Other work has suggested that VDR cross-regulates β-catenin [35] thus acting on the canonical Wnt pathway. These observations suggest vitamin D may act on both the canonical and non-canonical Wnt pathways. Much of the data on vitamin D signaling have been derived from cancer research, with several studies providing support for vitamin D/Wnt crosstalk [36, 37, 38]. There has been a growing understanding interactions between the Wnt and TGF pathways, and it is possible that vitamin D can play a role in modulating this relationship, thus demonstrating a more nuanced role in maintaining the pro- and anti-fibrotic balance within the CV system. To date, there are no published reports examining vitamin D signaling in human cardiac cells and further work will be needed to determine the precise mechanisms responsible for our observations in cardiac cells.

This study is limited in a number of aspects. Notably we did not observe significant downregulation of matrix remodeling proteins associated with TGFβ_1_ signaling other than αSMA. It is possible other regulatory mechanisms are involved in the expression of these proteins. Further, we did not elucidate the precise mechanism involved in the anti-fibrotic effects of vitamin D and more studies will be required in this regard. Our observational data regarding fibrosis in end-stage HF does not provide evidence of a causative role of vitamin D, particularly given the fluctuations in vitamin D that can occur seasonally and in response to lifestyle changes, and the extended time frame involved in myocardial remodeling. It is possible that chronic vitamin D deficiency may promote a pro-fibrotic remodeling program. In this light, while our data contribute to an existing understanding vitamin D's role in the CV system, continued work will be required to better define the complex role this hormone plays in health and disease.

Myocardial fibrosis is a critical contributor to progressive remodeling and the development of HF. Currently there are no available therapeutic agents which target this process [39]. Growing evidence of vitamin D's anti-fibrotic actions may indicate supplementation efforts in the setting of HF, or lead to the development of novel non-calcemic VDR agonists to target VDR signaling for treatment of pathological fibrosis in cardiac disease.

## Supporting Information

S1 File HCF-av *in vitro* raw data. (ZIP)

S2 File HF cohort fibrosis raw data. (DOCX)
