# Supplementary figures and images for: 1,25 Dihydroxyvitamin D3 Inhibits TGFβ1-Mediated Primary Human Cardiac Myofibroblast Activation
Source: PLoS One. 2015 Jun 10;10(6):e0128655. doi: 10.1371/journal.pone.0128655 (PMC4462580; doi:10.1371/journal.pone.0128655)

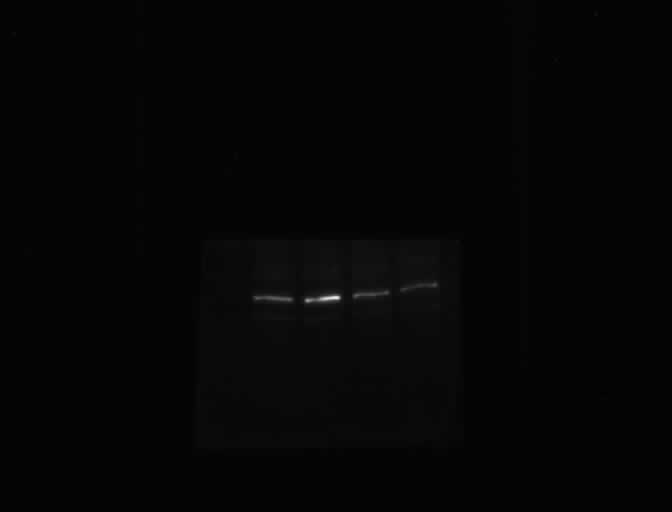

Supplement: S1 File — (ZIP) [file pone.0128655.s001.zip › Supplementary Data/WB images/Figure 1/Figure 1 - aSMA 41s.jpg]

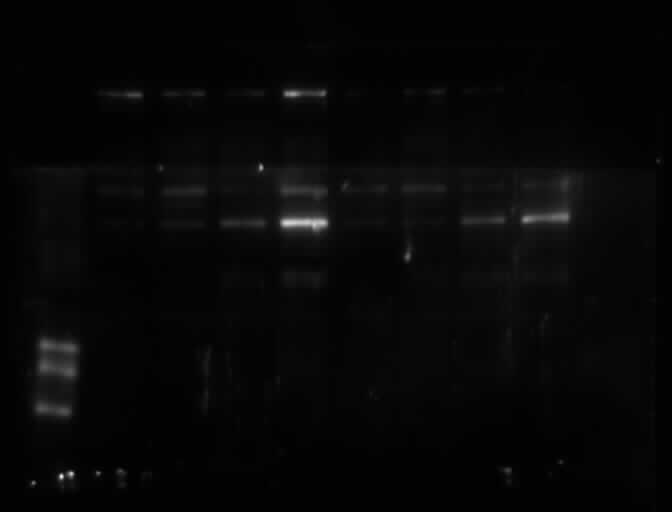

Supplement: S1 File — (ZIP) [file pone.0128655.s001.zip › Supplementary Data/WB images/Figure 1/Figure 1 - CYP24 5s.jpg]

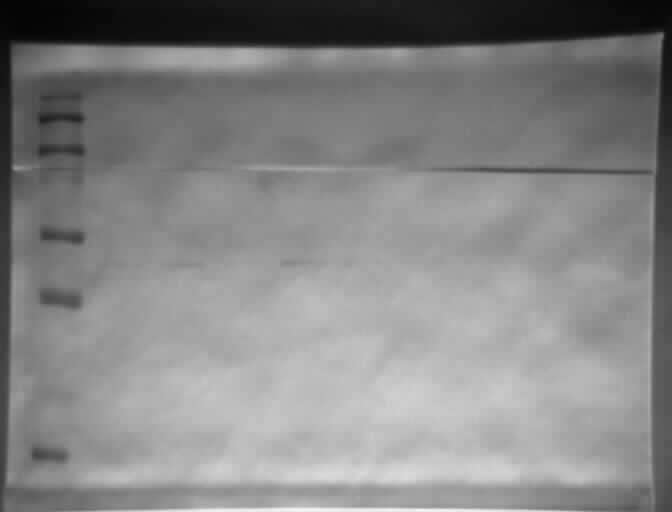

Supplement: S1 File — (ZIP) [file pone.0128655.s001.zip › Supplementary Data/WB images/Figure 1/Figure 1 - CYP24 white.jpg]

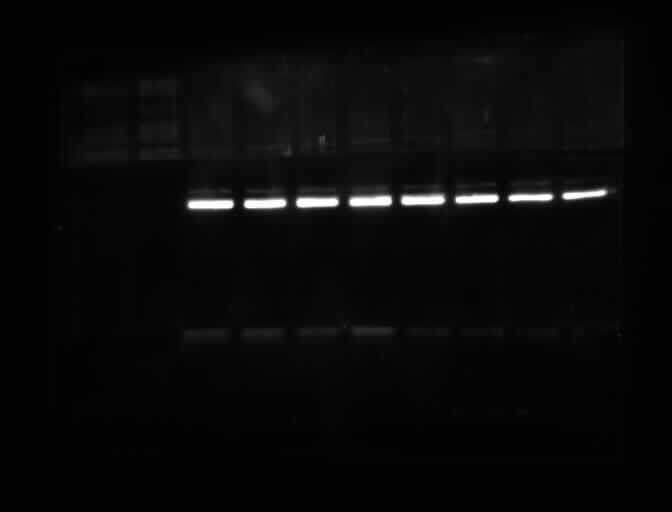

Supplement: S1 File — (ZIP) [file pone.0128655.s001.zip › Supplementary Data/WB images/Figure 1/Figure 1 - DDR2_VDR 15sec.jpg]

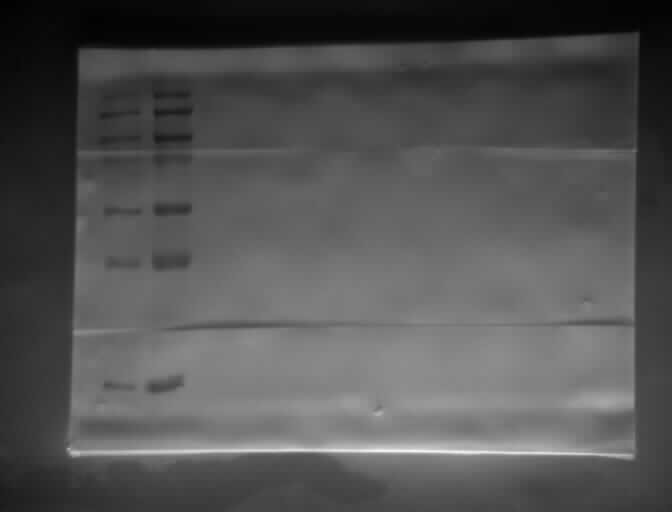

Supplement: S1 File — (ZIP) [file pone.0128655.s001.zip › Supplementary Data/WB images/Figure 1/Figure 1 - DDR2_VDR white.jpg]

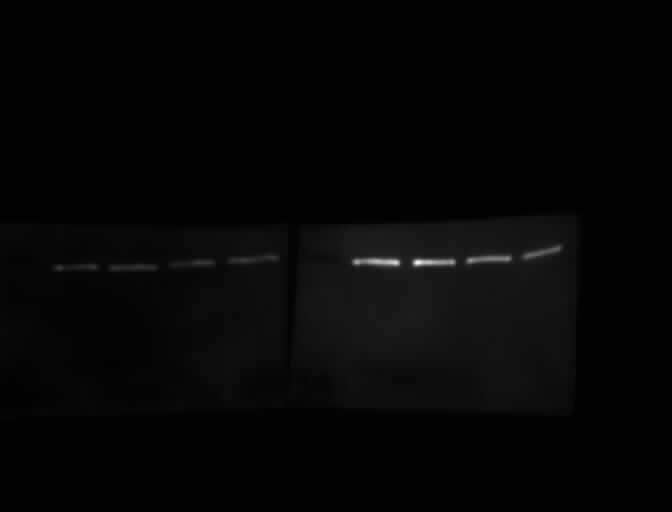

Supplement: S1 File — (ZIP) [file pone.0128655.s001.zip › Supplementary Data/WB images/Figure 1/Figure 1 - GAPDH 6S.jpg]

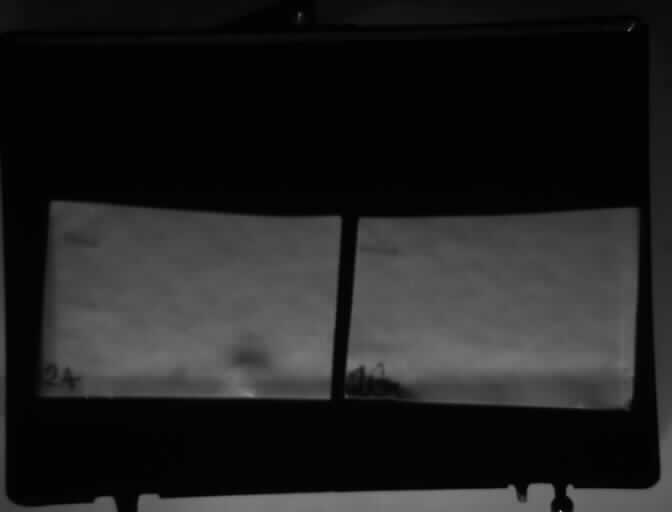

Supplement: S1 File — (ZIP) [file pone.0128655.s001.zip › Supplementary Data/WB images/Figure 1/Figure 1 - GAPDH WHITE.jpg]

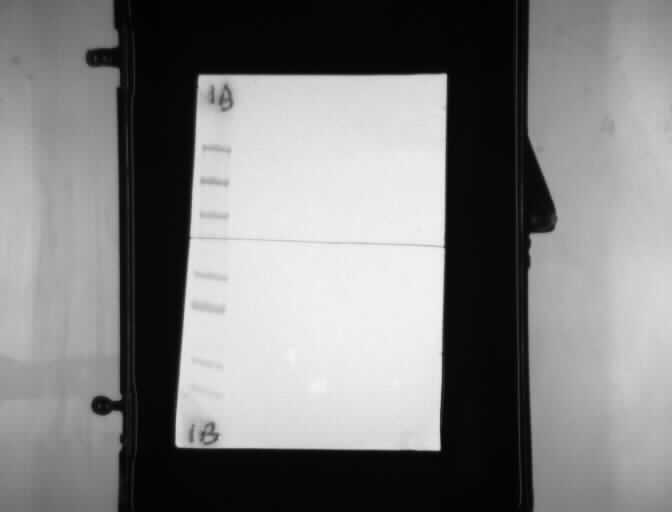

Supplement: S1 File — (ZIP) [file pone.0128655.s001.zip › Supplementary Data/WB images/Figure 1/Figure 1- aSMA white.jpg]

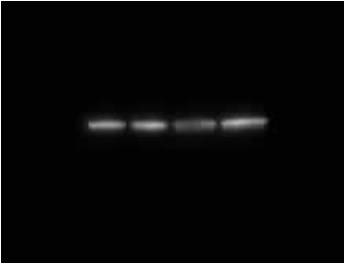

Supplement: S1 File — (ZIP) [file pone.0128655.s001.zip › Supplementary Data/WB images/Figure 4/Fig 4 - GAPDH 24hr.jpg]

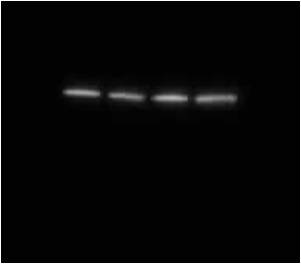

Supplement: S1 File — (ZIP) [file pone.0128655.s001.zip › Supplementary Data/WB images/Figure 4/Fig 4 - GAPDH 48hr.jpg]

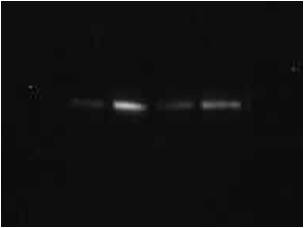

Supplement: S1 File — (ZIP) [file pone.0128655.s001.zip › Supplementary Data/WB images/Figure 4/Fig 4 - pSMAD 24hr.jpg]

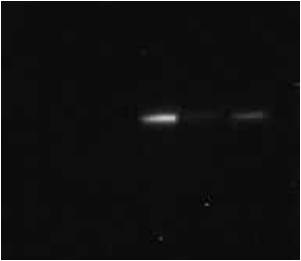

Supplement: S1 File — (ZIP) [file pone.0128655.s001.zip › Supplementary Data/WB images/Figure 4/Fig 4 - pSMAD 48hr.jpg]
