# Supplementary material for: 1,25 Dihydroxyvitamin D3 Inhibits TGFβ1-Mediated Primary Human Cardiac Myofibroblast Activation
Source: PLoS One. 2015 Jun 10;10(6):e0128655. doi: 10.1371/journal.pone.0128655 (PMC4462580; doi:10.1371/journal.pone.0128655)
Supplement: Supplementary file 4 [file pone.0128655.s002.docx]

Serum 25(OH)D (nmol/L)	% Fibrotic Area					Demographics	
	Field 1	Field 2	Field 3	Field 4	Field 5	Sex	Age
22	20.52937	31.57144	11.06376	17.76953	30.01885	M	47
29	8.863533	6.248291	9.0721	29.96091	23.65453	M	26
40	15.66519	25.69427	28.22482	19.07043	10.71784	M	20
45	13.63392	11.21107	23.20135	12.32377	41.68554	M	62
45	31.13555	41.31682	23.63168	21.36038	9.50881	M	53
48	36.72467	13.75848	16.38663	20.52045	19.92314	M	43
50	36.44758	18.57042	10.00348	12.13317	55.03253	F	40
51	5.675332	6.078374	10.51914	9.300563	10.30983	F	40
51	19.09624	27.25263	17.48894	17.52139	31.31324	M	58
61	48.65091	22.59869	8.113893	35.52103	31.03285	M	56
62	5.862374	2.932006	3.746619	3.134581	3.582673	M	60
63	6.008563	9.466484	10.74821	7.643382	8.164904	M	62
67	14.54508	13.79957	12.45473	23.42295	12.27812	M	62
67	10.55951	14.67067	8.935805	16.56209	18.41428	M	54
68	7.479847	14.36964	3.8038	7.966495	8.543425	M	26
70	9.324467	14.62915	9.479581	16.56901	15.09174	M	65
72	9.17756	34.1633	11.80355	14.52678	10.57601	M	64
72	13.30799	8.609516	4.340705	9.008915	10.62915	F	64
75	15.69186	8.283648	16.5803	19.25617	7.703598	F	58
75	6.785382	21.57152	6.715221	11.59561	8.095267	M	60
84	4.18003	10.04684	45.29436	30.54292	6.861962	M	61
94	7.268057	9.916003	7.677235	13.81782	7.672192	M	62
121	7.515695	7.598834	5.09831	3.704336	9.102383	F	66
